# Risk factors for early postoperative complications after D3 dissection for stage II or III colon cancer: Supplementary analysis of a multicenter randomized controlled trial in Japan (JCOG0404)

**DOI:** 10.1002/ags3.12246

**Published:** 2019-04-04

**Authors:** Yusuke Nishizawa, Tomonori Akagi, Masafumi Inomata, Hiroshi Katayama, Junki Mizusawa, Seiichiro Yamamoto, Masaaki Ito, Tadahiko Masaki, Masahiko Watanabe, Yasuhiro Shimada, Seigo Kitano

**Affiliations:** ^1^ Division of Gastroenterological Surgery Saitama Cancer Center Kitaadachi‐gun Saitama Japan; ^2^ Faculty of Medicine, Gastroenterological and Pediatric Surgery Oita University Yufu Oita Japan; ^3^ JCOG Operations Office National Cancer Center Hospital Tokyo Japan; ^4^ JCOG Data Center National Cancer Center Hospital Tokyo Japan; ^5^ Department of Surgery Hiratsuka City Hospital Hiratsuka Kanagawa Japan; ^6^ Department of Colorectal Surgery National Cancer Center Hospital East Kashiwa Chiba Japan; ^7^ Department of Surgery Kyorin University Tokyo Japan; ^8^ Department of Surgery Kitasato University School of Medicine Sagamihara Kanagawa Japan; ^9^ Department of Medical Oncology Kochi Health Sciences Center Kochi Japan; ^10^ Oita University Oita Japan

**Keywords:** colon cancer, D3, multivariate analysis, postoperative complication, risk factor

## Abstract

**Objective:**

To determine risk factors for early postoperative complications after D3 dissection for stage II/III colon cancer.

**Background:**

Identification of risk factors for postoperative complications is essential in patients surgically treated for colon cancer. The Japan Clinical Oncology Group (JCOG) conducted a randomized controlled trial, JCOG0404, to confirm the non‐inferiority of laparoscopic surgery (LAP) to open surgery (OP) with D3 dissection for stage II/III colon cancer. This supplementary analysis was made to assess risk factors for surgery requiring D3 dissection using data from JCOG0404.

**Methods:**

Proportion of postoperative complications of any grade (CTCAE ver. 3.0) until first discharge and risk factors for the most frequent complications were analyzed by univariable and multivariable analysis.

**Results:**

Among 1057 randomized patients treated between October 2004 and March 2009, 520 patients with OP and 525 patients with LAP were analyzed. Overall postoperative complications of all grades occurred in 190 patients (18.2%). Multivariable analysis showed that the risk factors for overall early postoperative complications were OP itself (odds ratio [OR] 2.01, 95% confidence interval [CI]: 1.38‐2.91, *P* = 0.0003) and operation time of >240 minutes (OR 1.94, 95% CI: 1.24‐3.02, *P* = 0.0036). The most frequent adverse event was wound complication (50/1045, 4.8%). In the univariable analysis, reconstruction, greater blood loss, OP, and higher body mass index were significantly associated with wound complication.

**Conclusion:**

Open surgery and longer operation time of >240 minutes were significant risk factors for postoperative complications. LAP surgery and shorter operation time could contribute to fewer postoperative complications in patients undergoing colectomy with D3 dissection. (Japan Clinical Oncology Group study JCOG 0404: NCT00147134/UMIN‐CTR: C000000105.)

## 1 INTRODUCTION

Preoperative identification and evaluation of risk factors for postoperative complications of colon cancer patients who undergo surgery would assist with informed consent for treatment, consideration of treatment options, and identification of high‐risk cases for special and possibly multidisciplinary attention. However, few reports have evaluated such risk factors of colon cancer surgery requiring D3 lymph node dissection, and those that have were small‐scale and retrospective studies. Additionally, risk factors such as age and long operation time did not always conform to each result.^1^, ^2^

JCOG0404 was a randomized controlled trial (RCT) conducted by the Colorectal Cancer Study Group of the Japan Clinical Oncology Group (JCOG) to confirm the non‐inferiority of laparoscopic surgery (LAP) in comparison with open surgery (OP) for patients with stage II/III colon cancer in terms of overall survival (OS) according to current practices. The surgical treatment of these two groups in the present study required D3 dissection equivalent to complete mesocolic excision with central vascular ligation.^3^ Additionally, JCOG0404 enrolled more than 1000 patients, making it one of the largest RCT for patients with colon cancer requiring D3 dissection in Japan.

We aimed to identify the risk factors for postoperative early complications in patients with stage II and III colon cancer by exploratory analyses using the data from JCOG0404. To the best of our knowledge, the present study is the first to evaluate risk factors for early postoperative complications after surgery requiring D3 dissection from prospectively collected data.

## 2 MATERIALS AND METHODS

### 2.1 Summary of JCOG0404

Eligibility criteria of JCOG0404 included histologically proven colon cancer with histologically confirmed adenocarcinoma, signet ring cell carcinoma, or adenosquamous carcinoma; tumor located in the cecum or ascending, sigmoid, or rectosigmoid colon; T3 or deeper lesion without involvement of other organs, N0‐2 and M0; tumor size ≤8 cm; and age 20‐75 years. Only accredited surgeons carried out surgery as an operator or instructor. Surgery required D3 lymph node dissection (details described below). Primary endpoint was OS. Non‐inferiority of LAP with D3 dissection to OP for OS was not confirmed for stage II/III colon cancer.

JCOG0404 is registered with UMIN Clinical Trials Registry, number C000000105, and ClinicalTrials.gov, number NCT00147134. Details of JCOG0404 were reported elsewhere.^4^, ^5^

### 2.2 Operative methods of D3 dissection

For right‐sided tumors, the vascular pedicles were divided at their origin, and the draining lymph nodes along the superior mesenteric vein were removed. For left‐sided tumors, removal of lymph nodes at the root of the inferior mesenteric artery was carried out along with high ligation or with preservation of the left colic artery and ligation of the root of the superior rectal artery.

### 2.3 Endpoints and statistical considerations

Adverse events were evaluated according to CTCAE 3.0. Postoperative mortality and morbidity were respectively defined as death from any cause and any adverse event of grade 1 or higher including anastomotic leakage, paralytic ileus, bowel obstruction, urinary tract infection, and wound complication occurring within 30 days after surgery. Background characteristics of the patients with postoperative complications were compared with those without postoperative complications. From the clinical point of view, the following eight variables were selected for univariable analysis: (i) surgical procedure (ileocolic resection or right colectomy vs sigmoid colectomy vs anterior resection vs others); (ii) reconstruction (hand sewing vs functional end‐to‐end anastomosis vs stapled anastomosis vs others); (iii) operation time (<240 minutes vs ≥240 minutes); (iv) blood loss (<300 mL vs ≥300 mL); (v) combined resection (absent vs present); (vi) gender (female vs male); (vii) treatment arm (OP vs LAP); and (viii) body mass index (BMI) (≤20 vs 20 < BMI ≤ 25 vs >25 kg/m^2^). Univariable analysis was carried out by Fisher's exact test to compare treatment arms in terms of operative morbidity and mortality. To investigate risk factors for postoperative morbidity, univariable and multivariable logistic regression analysis was done and the odds ratio (OR) and its 95% confidence interval (CI) for postoperative morbidity were estimated. For wound complications and leakage, only univariable logistic regression analysis was carried out as a result of insufficient events to perform multivariable analysis. The population included in this analysis was defined as those patients who received the assigned surgery; hence, patients who did not receive the assigned surgery were excluded from this analysis. A two‐sided *P*‐value of <0.05 was considered statistically significant. All statistical analyses were carried out using SAS ver. 9.2 or higher.

## 3 RESULTS

**Figure 1:**
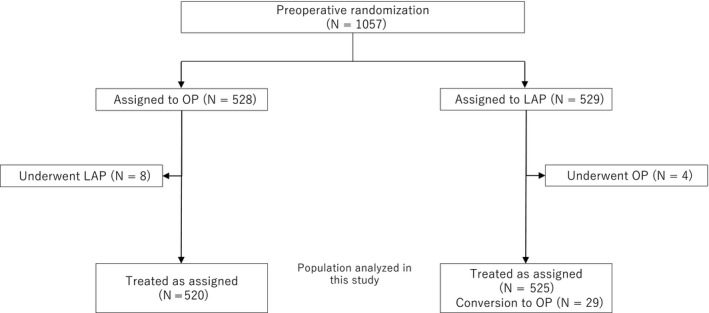
Total of 1057 patients from 30 Japanese centers were randomized to the open surgery (OP) group (528 patients) or to the laparoscopic surgery (LAP) group (529 patients). Treatment assignment was balanced with respect to baseline characteristics. In the 528 patients assigned to OP, 8 patients underwent LAP. In contrast, among the 529 patients assigned to LAP, 4 patients underwent OP. Finally, 520 patients underwent OP and 525 patients underwent LAP, and the results were compared and analyzed

Between October 2004 and March 2009, 1057 patients from 30 Japanese centers were randomized to the OP group (528 patients) and LAP group (529 patients). Treatment assignment was balanced with respect to baseline characteristics. In the 528 patients assigned to OP, eight patients underwent LAP. In contrast, among the 529 patients assigned to LAP, four patients underwent OP. Finally, 520 patients underwent OP and 525 patients underwent LAP, and the results were compared and analyzed (Figure 1).

Table 1 shows operative morbidity and mortality of all patients in the OP and LAP groups. Overall postoperative complications of all grades occurred in 190 patients (18.2%). Perioperative mortality was observed in one (0.2%) patient in the OP group because of myocardial infarction. Conversion to OP was necessary in 29 (5.5%) of the 525 LAP group patients. The most common reason for conversion was an indicated conversion: tumor invasion to adjacent structures (9 [31%] patients), peritoneal dissemination (4 [14%] patients), synchronous ascending colon cancer found during surgery (1 [3%] patient), and synchronous hepatic metastasis found during LAP that was removed by conversion to OP (1 [3%] patient). The other reasons for conversion were reported elsewhere.^5^

**Table 1:** Operative morbidity and mortality of all patients in the OP and LAP groups

	Grade	OP, n = 520	LAP, n = 525	*P* value
Any overall complication (except for high fever)	G0	405 (77.9%)	450 (85.7%)	0.0013
	G1‐	115 (22.1%)	75 (14.3%)	
Upper respiratory infection	G0	519 (99.8%)	524 (99.8%)	1.00
	G1‐	0 (0%)	1 (0.2%)	
Urinary tract infection	G0	517 (99.4%)	523 (99.6%)	0.69
	G1‐	3 (0.6%)	2 (0.4%)	
Anastomotic leakage	G0	502 (96.5%)	506 (96.4%)	1.00
	G1‐	18 (3.5%)	19 (3.6%)	
Bowel obstruction	G0	515 (99.0%)	521 (99.2%)	0.50
	G1‐	5 (1.0%)	4 (0.8%)	
Wound complication	G0	481 (92.5%)	514 (97.9%)	<0.001
	G1‐	39 (7.5%)	11 (2.1%)	
Mortality	Absent	519 (99.8%)	525 (100%)	0.50
	Present	1 (0.2%)	0 (0.0%)	

LAP, laparoscopic surgery; OP, open surgery.

Table 2 shows the results of the univariable and multivariable analyses of the risk factors for overall complications. Among the eight variables (procedure, reconstruction, operation time, blood loss, combined resection, gender, BMI, and surgical group), operation time of over 240 minutes, blood loss of more than 300 mL, male gender, and OP itself were significantly associated with postoperative complications in the univariable analyses. Multivariable analyses excluding one missing patient showed that the risk factors for all grades of early postoperative complications were operation time of over 240 minutes (OR 1.94, 95% CI: 1.24‐3.02, *P* = 0.0036) and OP group (OR 2.01, 95% CI: 1.38‐2.91, *P* = 0.0003).

**Table 2:** Risk factors for overall complications

Risk factor	Complication (−)	Complication (+)	Univariable analysis	*P* value	Multivariable analysis	*P* value
			Odds ratio (95% CI)		Odds ratio (95% CI)	
Surgical procedure						
ICR or right colectomy	248	50 (16.8%)	1		1	
Sigmoid colectomy	372	68 (15.5%)	0.91 (0.61‐1.35)	0.63	0.63 (0.31‐1.30)	0.21
Anterior resection	221	70 (24.1%)	1.57 (1.05‐2.36)	0.029	0.83 (0.37‐1.87)	0.65
Others	14	2 (12.5%)	0.71 (0.16‐3.22)	0.66	0.44 (0.08‐2.34)	0.34
Reconstruction (1 pt. missing)						
Hand‐sewing	73	9 (11.0%)	1		1	
FETE	250	47 (15.8%)	1.53 (0.71‐3.26)	0.28	1.39 (0.62‐3.11)	0.43
Stapled anastomosis	512	127 (19.9%)	2.01 (0.98‐4.13)	0.057	2.08 (0.89‐4.86)	0.091
Other	19	7 (26.9%)	2.99 (0.99‐9.06)	0.053	3.93 (1.25‐12.33)	0.019
Operation time (min)						
<240	694	137 (16.5%)	1		1	
≥240	161	53 (24.8%)	1.67 (1.16‐2.39)	0.005	1.94 (1.24‐3.02)	0.004
Blood loss (mL)						
<300	813	164 (16.8%)	1		1	
≥300	42	26 (38.2%)	3.07 (1.83‐5.15)	<0.0001	1.76 (0.99‐3.13)	0.055
Combined resection						
Absent	803	174 (17.8%)	1		1	
Present	52	16 (23.5%)	1.42 (0.79‐2.55)	0.24	1.19 (0.64‐2.23)	0.58
Gender						
Female	387	71 (15.5%)	1		1	
Male	468	119 (20.3%)	1.39 (1.00‐1.92)	0.048	1.16 (0.82‐1.64)	0.41
BMI (kg/m^2^)						
BMI ≤ 20	150	24 (13.8%)	1		1	
20 < BMI ≤ 25	503	113 (18.3%)	1.40 (0.87‐2.26)	0.16	1.26 (0.77‐2.07)	0.36
BMI > 25	202	53 (20.8%)	1.64 (0.97‐2.78)	0.066	1.37 (0.79‐2.39)	0.26
Treatment arm						
LAP	450	75 (14.3%)	1		1	
OP	405	115 (22.1%)	1.70 (1.24‐2.35)	0.001	2.01 (1.38‐2.91)	<0.001

BMI, body mass index; CI, confidence interval; FETE, functional end‐to‐end anastomosis; ICR, ileocecal resection; LAP, laparoscopic surgery; OP, open surgery.

Wound complication was the most frequent postoperative complication among all grades (50/1045, 4.8%). There was one and no Grade 3 complications of intraperitoneal abscess in the OP and LAP groups, respectively. Univariable analysis showed that reconstruction (others: triangle anastomosis in 25 patients and stoma creation in 1 patient), greater blood loss, OP itself, and higher BMI were significantly associated with wound‐related complications (Table 3).

**Table 3:** Risk factors for wound complications

Risk factor	Complication (−)	Complication (+)	Univariable analysis	*P* value
			Odds ratio (95% CI)	
Surgical procedure				
ICR or right colectomy	285	13 (4.4%)	1	
Sigmoid colectomy	420	20 (4.5%)	1.04 (0.51‐2.13)	0.91
Anterior resection	275	16 (5.5%)	1.28 (0.60‐2.70)	0.52
Other	15	1 (6.3%)	1.46 (0.18‐11.93)	0.72
Reconstruction (1 pt. missing)				
Hand‐sewing	79	3 (3.7%)	1	
FETE	284	13 (4.4%)	1.21 (0.34‐4.34)	0.77
Stapled anastomosis	610	29 (4.5%)	1.25 (0.37‐4.21)	0.72
Other	21	5 (19.2%)	6.27 (1.39‐28.78)	0.017
Operation time (min)				
<240	788	43 (5.2%)	1	
≥240	207	7 (3.3%)	0.62 (0.28‐1.40)	0.25
Blood loss (mL)				
<300	935	42 (4.3%)	1	
≥300	60	8 (11.8%)	2.97 (1.33‐6.61)	0.008
Combined resection				
Absent	931	46 (4.7%)	1	
Present	64	4 (5.9%)	1.27 (0.44‐3.63)	0.66
Gender				
Female	436	22 (4.8%)	1	
Male	559	28 (4.8%)	0.99 (0.56‐1.76)	0.98
BMI (kg/m^2^)				
BMI ≤ 20	172	2 (1.1%)	1	
20 < BMI ≤ 25	580	36 (5.8%)	5.34 (1.27‐22.39)	0.022
BMI > 25	243	12 (4.7%)	4.25 (0.94‐19.22)	0.060
Treatment arm				
LAP	514	11 (2.1%)	1	
OP	481	39 (7.5%)	3.79 (1.92‐7.46)	<0.001

BMI, body mass index; CI, confidence interval; FETE, functional end‐to‐end anastomosis; ICR, ileocecal resection; LAP, laparoscopic surgery; OP, open surgery.

Anastomotic leakage occurred in 37 patients (3.5%). There was no difference in its occurrence between the OP group (18; 3.5%) and the LAP group (19; 3.6%). Univariable analysis showed that gender, operation time, and type of procedure were significantly associated with anastomotic leakage (Table 4).

**Table 4:** Risk factors for leakage

Risk factor	Complication (‐)	Complication (+)	Univariable analysis	*P* value
			Odds ratio (95% CI)	
Surgical procedure				
ICR or right colectomy	297	1 (0.3%)	1	
Sigmoid colectomy	425	15 (3.4%)	10.48 (1.38‐84.79)	0.023
Anterior resection	270	21 (7.2%)	23.10 (3.09‐172.89)	0.002
Other	16	0 (0%)	NA	NA
Reconstruction (1 pt. missing)				
Hand‐sewing	81	1 (1.2%)	1	
FETE	295	2 (0.7%)	0.55 (0.05‐6.13)	0.63
Stapled anastomosis	605	34 (5.3%)	4.55 (0.61‐33.71)	0.14
Other	26	0 (0%)	NA	NA
Operation time (min)				
<240	809	22 (2.6%)	1	
≥240	199	15 (7.0%)	2.77 (1.41‐5.44)	0.003
Blood loss (mL)				
<300	945	32 (3.3%)	1	
≥300	63	5 (7.4%)	2.34 (0.88‐6.22)	0.087
Combined resection				
Absent	943	34 (3.5%)	1	
Present	65	3 (4.4%)	1.28 (0.38‐4.28)	0.69
Gender				
Female	450	8 (1.7%)	1	
Male	558	29 (4.9%)	2.92 (1.32‐6.46)	0.008
BMI (kg/m^2^)				
BMI ≤ 20	168	6 (3.4%)	1	
20 < BMI ≤ 25	597	19 (3.1%)	0.89 (0.35‐2.27)	0.81
BMI > 25	243	12 (4.7%)	1.38 (0.51‐3.76)	0.53
Treatment arm				
LAP	506	19 (3.6%)	1	
OP	502	18 (3.6%)	0.96 (0.50‐1.84)	0.89

BMI, body mass index; CI, confidence interval; FETE, functional end‐to‐end anastomosis; ICR, ileocecal resection; LAP, laparoscopic surgery; NA, not available; OP, open surgery.

## 4 DISCUSSION

Our analysis showed that the OP procedure and operation time of over 240 minutes were significant risk factors for overall postoperative complications of all grades. LAP would appear to be more suitable than OP from the point of view of early postoperative complications for stage II/III colon cancer patients undergoing D3 dissection.

In previous reports of large‐scale RCT for colon cancer that compared OP with LAP, Lacy et al^6^ reported that there were more complications in OP than in LAP, as in the present study, whereas other reports^7^, ^8^, ^9^ showed that there were no differences between the two. The conversion rate from LAP to OP surgery in the present study was lower than that in previous RCT. The conversion rate was 5.5% (29/525), whereas it was 21% (90/435) in the COST trial,^7^ 29% (143/488) in the CLASICC trial,^8^ and 19% (102/534) in the COLOR trial.^9^ Generally, a lower conversion rate means fewer adverse events because the reasons for conversion were based on intraoperative complications, progression of tumors including invasion of adjacent structures, and technical difficulty as a result of huge tumor size and to control bleeding. We speculated that the data from the present study and from the study of Lacy et al showing conversion rates of approximately 10% or less might lead to better short‐term outcomes in LAP than in OP. Therefore, we might obtain better short‐term outcomes if we can choose the appropriate indication for LAP and improve or use additional techniques to avoid conversion.

So far, several reports have shown the risk factors for overall complications to be male gender,^10^ cancer stage,^10^ BMI,^10^ visceral fat area,^11^ age,^12^ OP^12^, ^13^ and blood loss.^13^ The OP procedure was also shown to be a risk factor in our analysis in comparison with LAP. However, male gender, BMI, and blood loss were not risk factors for overall postoperative complications in our analysis. The present study is the first, to our knowledge, to show that in addition to the OP procedure, operation time of over 240 minutes was a risk factor for overall postoperative complications. We speculated that longer operation time might be broadly affected by tumor factors, including larger size and invasion to adjacent structures, and patient factors, including BMI and vascular comorbidities, despite not identifying each significant factor for complications individually, which could lead to a higher possibility of postoperative complications. This is why the present study did not need to take into consideration the surgical learning curve as a potential risk factor because the JCOG0404 study chairman certifies surgeons at each participating institution according to the following criteria. For OP, surgeons must have experience of 30 or more OP colectomies; for LAP, surgeons must have experience of 30 or more cases each of OP and LAP colectomies; and surgeons in the LAP arm must be certified according to the Endoscopic Surgery Skill System by the Japan Society for Endoscopic Surgery.

Ishihara et al^10^ reported that stage as a tumor factor and hypertension as a patient factor were significantly associated with an increased incidence of noninfectious complications. They also showed that operation time had a significant relation in patients with BMI >25 kg/m^2^, cancers in the rectum, and cerebrovascular disease, but operation time was not related with stage. We did not include age as a risk factor because all patients were between 20 and 75 years old. The studies of Kim and Kim^14^ and Ragg et al,^15^ respectively, showed that age greater than 90 years old and age greater than 75 years old to be one risk factor for overall postoperative complications.

An important point of difference between the present study and the previous RCT^6^, ^9^, ^16^, ^17^ is that surgical treatment of the two groups in the present study required D3 dissection for stage II/III colon cancer equivalent to complete mesocolic excision with central vascular ligation.^3^ To the best of our knowledge, the present study is the first to evaluate risk factors for postoperative complications after surgery requiring D3 dissection from prospectively collected data. Japan has had a long history of using D3 dissection according to the General Rules for Clinical and Pathological Studies on Cancer of the Colon, Rectum and Anus (first edition, 1977).^18^ D3 dissection was carried out to unify the surgical maneuver in the JCOG0404 trial. For quality control, the trial ensured that the surgeries were carried out by accredited surgeons, and a central review of each surgery was done on the basis of photographs obtained during the procedure. For the JCOG0404 trial, Nakajima et al reported that for right‐sided tumors, the proportion of D3 dissections was 98.5% (131/133) in OP and 100% (136/136) in LAP, and for left‐sided tumors, the proportion was 97.9% (322/329) in OP and 98.2% (320/326) in LAP, as evaluated by central review.^19^ Because quality of surgery is one of the important factors of surgery‐related adverse events, in terms of the proportion of D3 dissections carried out by accredited surgeons, the quality of surgery in the present study was high. Thus, we consider the risk factors derived in the present study to be reliable and reproducible.

There are limitations in the present study. The inclusion criteria of JCOG0404 did not include disease in the transverse or descending colon, which would have required high‐level laparoscopic skills. The incidences of wound complications and anastomotic leakage were insufficient for multivariable analysis. However, 37 patients (3.5%) developed anastomotic leakage in our study. With anastomotic leakage being one of the most critical complications, these are not satisfactory data. Thus, further investigation is necessary to identify the risk factors for anastomotic leakage.

In conclusion, our analysis from prospectively collected data of D3 dissection for stage II/III colon cancer indicated that the risk factors for overall postoperative complications were the OP procedure and operation time of over 240 minutes. LAP and shorter operation time may contribute to a decrease in postoperative complications for patients undergoing colectomy with D3 dissection.

## DISCLOSURE

Conflicts of Interest: Authors declare no conflicts of interest for this article.
